# Long-term effects of denosumab on bone mineral density and turnover markers in patients undergoing hemodialysis

**DOI:** 10.1007/s00774-024-01505-7

**Published:** 2024-03-21

**Authors:** Kazuhiko Kato, Tatsuhiro Yaginuma, Arisa Kobayashi, Akio Nakashima, Ichiro Ohkido, Takashi Yokoo

**Affiliations:** 1 Division of Nephrology and Hypertension, Department of Internal Medicine, The Jikei University School of Medicine, 3-25-8 Nishi-Shimbashi, Minato-Ku, Tokyo 105-8461 Japan; 2 Division of Molecular Epidemiology, The Jikei University School of Medicine, Tokyo, Japan; 3 Division of Nephrology, Keijin Hospital, Tokyo, Japan; 4 Human Health Sciences, Kyoto University Graduate School of Medicine, Kyoto, Japan

**Keywords:** Bone mineral density, Bone turnover markers, Denosumab, Hemodialysis

## Abstract

**Introduction:**

Denosumab, a fully human anti-RANKL monoclonal antibody, is a widely used osteoporosis treatment that is increasingly being used in patients undergoing dialysis; however, its long-term efficacy and safety in these patients remain unknown.

**Materials and methods:**

This observational study comprised individuals aged ≥ 20 years undergoing hemodialysis and receiving denosumab. After denosumab administration, we analyzed the long-term changes in bone mineral density (BMD) and levels of bone turnover markers (BTMs) and calcium.

**Results:**

The study included 45 patients who have been receiving denosumab for a median duration of 3.8 (interquartile range, 2.5–6.7) years. Tartrate-resistant acid phosphatase 5b (TRACP-5b) levels decreased from a median of 595 (434–778) mU/dL at baseline to 200 (141–430) mU/dL after 6 months of denosumab administration (*P* < 0.001) and remained low thereafter. Similarly, bone-specific alkaline phosphatase (BAP) levels decreased from a median of 18.2 (15.9–25.8) μg/L at baseline to 12.4 (9.9–15.6) μg/L after 6 months (*P* < 0.001) and remained low thereafter. Meanwhile, BMD, as assessed with dual energy X-ray absorptiometry and measured at the distal 1/3 of the radius, did not decrease (0.465 ± 0.112 g/cm^2^ at baseline vs. 0.464 ± 0.112 g/cm^2^ after administration; *P* = 0.616). Regarding hypocalcemia, corrected calcium levels reached were the lowest at 7 days after administration and normalized within 30 days.

**Conclusion:**

The study showed long-term suppression of TRACP-5b and BAP levels and sustaining BMD after denosumab administration over an extended period in patients undergoing hemodialysis.

**Supplementary Information:**

The online version contains supplementary material available at 10.1007/s00774-024-01505-7.

## Introduction

Patients undergoing dialysis have a five-fold higher risk of experiencing fractures compared with the general population [1]; once a fracture occurs, their risk of death increases [2, 3]. Consequently, preventing fractures in patients undergoing dialysis not only improves their quality of life but also their prognosis. The pathogenesis of osteoporosis in patients on dialysis is more complex than that in the general population as impaired renal function leads to various pathologies, including anemia and chronic kidney disease-mineral and bone disorder (CKD-MBD), that also affect bone metabolism [4]. Exhaustive systematic reviews focusing on osteoporosis therapeutics in the chronic kidney disease (CKD) subset have yielded insufficient evidence of the effectiveness of each therapeutic agent within this group, and paradigms for fracture deterrence in patients undergoing dialysis are still in the early stages [5].

Denosumab, a human anti-RANKL monoclonal antibody, decreases bone resorption and is used to treat osteoporosis [6]. Several studies have indicated that denosumab restores bone mineral density (BMD) and prevents bone fractures, with a relative risk reduction of 40%–68% in the general population [4, 7–14]. The effect of denosumab is even more significant in patients with CKD, including those on dialysis, as it eliminates the need for volume regulation of renal function [10, 15]. Although several observational studies have been conducted on patients with CKD [14, 16–18], there is a lack of research investigating the long-term effects of denosumab on bone turnover markers (BTMs) and BMD within this group.

Currently, long-term efficacy and safety of denosumab in patients undergoing hemodialysis remain unknown, and the same is true for its long-term effects on BMD and bone mineral metabolism. A more comprehensive repository of information is warranted to establish denosumab as a preventive drug against fractures in patients undergoing hemodialysis. Therefore, we designed an observational study involving patients on hemodialysis receiving denosumab to determine changes in BMD and levels of BTMs and calcium (Ca).

## Materials and Methods

### Study design

In this observational retrospective study, we investigated changes in BMD and levels of BTMs and Ca in patients undergoing hemodialysis and receiving denosumab.

### Ethics approval

All procedures involving human participants were approved by the institutional review board of our hospital (approval number: 34–387 [11544]) and conducted in accordance with the ethical standards of the 1964 Helsinki Declaration and its later amendments. Due to the retrospective nature of this study, the need to obtain informed consent from patients was waived by the institutional review board.

### Data source and patient selection

Our investigation involved individuals aged ≥ 20 years who were undergoing hemodialysis in Keijin Hospital, a maintenance hemodialysis center in Tokyo, Japan. The attending physician made the decision for denosumab administration based on the Japanese guidelines for osteoporosis [19]. The chronological term for this research extended from December 2013 through December 2022, during which time data were gathered retrospectively over a median duration of 3.8 (interquartile range [IQR]: 2.5–6.7) years for each patient.

### Clinical parameters

Blood tests were performed at the beginning of the week before the start of hemodialysis. Levels of hemoglobin (g/dL), urea nitrogen (mg/dL), creatinine (mg/dL), albumin (Alb) (g/dL), Ca (mg/dL), phosphate (mg/dL), magnesium (mg/dL), alkaline phosphatase (U/L), intact parathyroid hormone (PTH) (pg/mL), tartrate-resistant acid phosphatase 5b (TRACP-5b) (mU/dL), and bone-specific alkaline phosphatase (BAP) (μg/L) were measured using standard commercial assays. For serum Ca levels, the corrected Ca (cCa) level was calculated as follows if the serum albumin level was < 4.0 mg/dL: cCa = Ca + 4.0—Alb. BMD was assessed by dual-energy X-ray absorptiometry (DXA; Dichroma Scan, DCS-600 EXV, Hitachi Aloka Medical, Tokyo, Japan) and measured at the distal 1/3 of the radius. Patients in this study underwent BMD measurements once a year, and measurements before denosumab administration and at the end of the observation period were evaluated. We also reviewed medications and comorbidities based on medical records.

### Statistical analyses

Data are presented as means ± standard deviations or medians with IQR. First, we showed changes in BMD and levels of BTMs, cCa, phosphate, and intact PTH using box-beard diagrams (point: median; box, IQR; beard, 95% confidence interval [CI]), and each change was tested using the paired-sample *t* test. Thereafter, associations between TRACP-5b, BAP, and intact PTH levels and decrease of cCa levels were evaluated by determining the correlation coefficient and illustrating scatter plots and regression lines. All tests performed during this study were two-sided, with a *P* value < 0.05 indicating statistical significance. A complete case analysis was performed when data were missing. All statistical analyses were performed using Stata version 15.1 (StataCorp LLC, College Station, TX, USA).

## Results

### Patient characteristics

The study included 45 patients who had available data on BMD and levels of BTMs and cCa after denosumab administration. Table 1 presents the characteristics of all patients in this study. The median (25–75%) patient age was 75 (range 70–80) years. Patients had been undergoing dialysis for a median of 55 (24–113) months. All patients received denosumab every 6 months during the observation period.

**Table 1:** Patient characteristics (*n* = 45)

Variable		
Age, years	75	(70–80)
Sex (female), *n* (%)	28	(62.2)
Comorbidities		
Dialysis vintage, months	55	(24–113)
Diabetes mellitus, *n* (%)	19	(42.2)
Past bone fracture, *n* (%)	19	(42.2)
Past parathyroidectomy, *n* (%)	2	(4.4)
Laboratory test measurements		
Hemoglobin, g/dL	10.7	± 1.1
Urea nitrogen, mg/dL	57.4	± 16.9
Creatinine, mg/dL	7.9	± 2.8
Albumin, g/dL	3.6	± 0.6
Calcium, mg/dL	8.6	± 0.6
Phosphate, mg/dL	4.5	(3.5–5)
Magnesium, mg/dL	2.5	(2.3–2.7)
Alkaline phosphatase, U/L	307	(252–391)
Intact PTH, pg/mL	89	(57–202)
Treatment		
Phosphate binder (calcium), *n* (%)	29	(64.4)
Phosphate binder (non-calcium), *n* (%)	13	(28.9)
Active vitamin D analogs, *n* (%)	41	(91.1)
Calcimimetics, *n* (%)	26	(57.8)

Continuous variables are summarized as means (± standard deviation) or medians (interquartile ranges). Categorical variables are shown as numbers of patients (%)

*PTH* parathyroid hormone

### Changes in BTM levels following denosumab administration

**Fig. 1:**
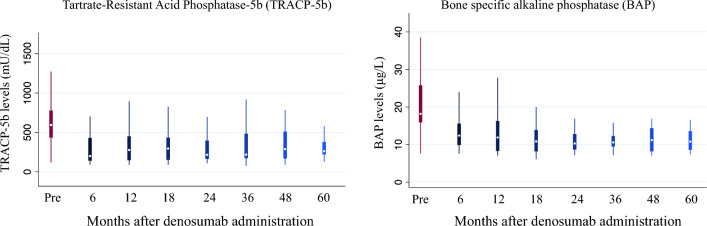
Changes in bone turnover markers after denosumab administration

Figure 1 shows the changes in TRACP5-b and BAP levels over time. TRACp-5b levels decreased from a median of 595 (434–778) mU/dL at baseline to 200 (141–430) mU/dL after 6 months of denosumab administration and remained low thereafter. Similarly, BAP levels decreased from a median of 18.2 (15.9–25.8) μg/L at baseline to 12.4 (9.9–15.6) μg/L after 6 months of administration and remained low thereafter. All post-administration TRACP5-b and BAP levels were significantly lower than the baseline levels (*P* < 0.001).

### Changes in BMD following denosumab administration

**Fig. 2:**
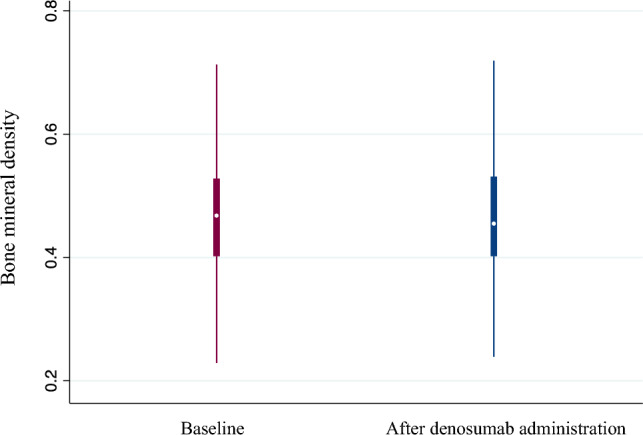
Changes in bone mineral density after denosumab administration

BMD did not significantly change before and after denosumab administration, from a mean BMD of 0.465 (± 0.112) g/cm^2 ^at baseline to a mean BMD of 0.464 (± 0.112) g/cm^2^ after administration (*P* = 0.616) (Fig. 2).

### Changes in cCa levels following denosumab administration

**Fig. 3:**
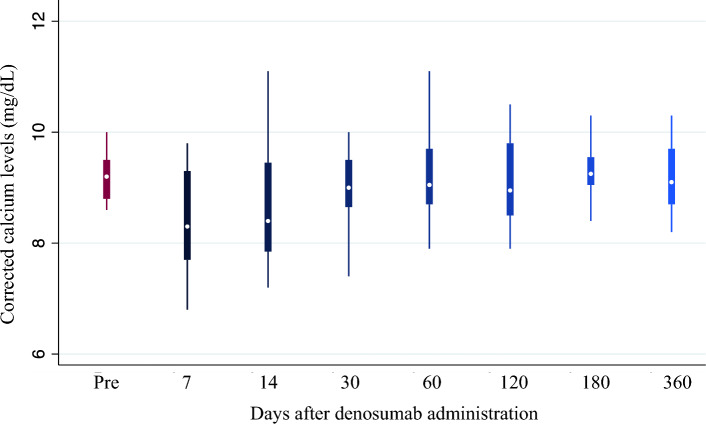
Changes in corrected calcium levels after denosumab administration

CCa levels were the lowest at a median of 8.3 (7.7–9.3) mg/dL on the seventh day after administration and subsequently increased to a median of 9.2 mg/dL, similar to the baseline levels (Fig. 3).

### Correlation between BTMs and cCa decreased

**Fig. 4:**
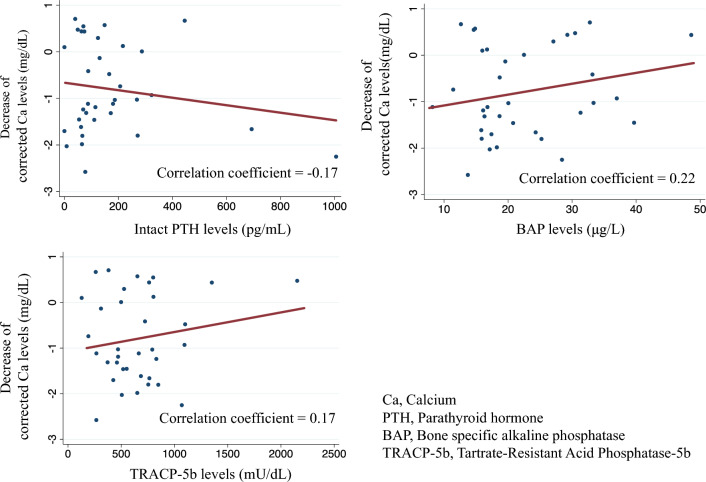
Correlations between each variable and decrease in corrected calcium levels on day 7

Figure 4 and Supplementary Fig. 1 show the correlation between intact PTH, BAP, and TRACP5-b levels and the cCa decrease at 7 days of administration, as well as the maximum decrease in cCa until 30 days of administration, respectively. The correlation coefficient between the decrease in cCa levels at day 7 and intact PTH is − 0.17, while it is 0.22 with BAP, and 0.17 with TRACP5-b. These coefficients change to − 0.22 for PTH, 0.19 for BAP, and 0.14 for TRACP5-b at 30 days. Hence, BTMs were not significantly correlated with lower cCa levels.

### Changes in phosphate and intact PTH levels

Supplementary Figs. 2 and 3 show the changes in phosphate and intact PTH levels. Phosphate levels were the lowest at a median of 3.5 (2.8–4.3) mg/dL on the seventh day after administration and subsequently increased to a median of 4.1 mg/dL, similar to baseline levels (Supplementary Fig. 2). Intact PTH levels did not change significantly after denosumab administration and generally remained less than 200 pg/mL (Supplementary Fig. 3).

## Discussion

In the present study, continuous denosumab administration rapidly reduced TRACP5-b and BAP levels, which remained low throughout the extended observation period. Meanwhile, BMD was maintained during the observation period. Regarding hypocalcemia, cCa levels reached their lowest at 7 days after denosumab administration and normalized within 30 days. Previous studies reporting changes in BTMs in patients undergoing hemodialysis after denosumab administration had a short follow-up period of 1 or 2 years [4, 16, 17], and they emphasized the need to determine the long-term trends in bone metabolism markers. Additionally, it was unknown whether suppression of bone turnover following denosumab administration was sustained over time. Thus, this study is the first to report that denosumab causes long-term suppression of BTMs.

Bone biopsy is the gold standard for assessing bone metabolism. However, it is invasive and complex to perform routinely in clinical practice. In contrast, measuring BTM levels is noninvasive and amenable to recurrent testing, facilitating longitudinal assessments of shifts in bone metabolism. Elevated TRACP5-b levels in the general population suggest increased bone resorption, making TRACP a widely used quantitative BTM [20]. Moreover, TRACP5-b is not influenced by renal function; hence, it is suitable for evaluating bone resorption in patients undergoing hemodialysis [20]. Notably, previous studies on denosumab in patients undergoing hemodialysis have reported a rapid decrease in TRACP5-b levels after administration [4, 16, 17].

BAP, which constitutes approximately half of the alkaline phosphatase derived from diverse tissues, is a crucial biomarker for bone formation assessment. BAP reportedly helps determine if bone formation is downregulated in the general population [21], and it is a beneficial marker in patients undergoing hemodialysis because it is not affected by renal function. Furthermore, BAP levels are significant predictors of fracture risk in patients undergoing hemodialysis [22]. Previous studies on denosumab in patients undergoing hemodialysis have also shown a rapid decrease in BAP levels after administration [4, 16, 17] similar to the present study, suggesting that the inhibition of bone resorption by denosumab concurrently leads to a reduction in bone formation.

Earlier reports in the general population have indicated that extended use of denosumab leads to the continuous suppression of TRACP5-b and BAP [23]. However, the long-term trends of these BTMs in patients on hemodialysis have yet to be explored. In this study, we showed for the first time that long-term administration of denosumab continuously suppressed TRACP5-b and BAP in patients undergoing hemodialysis.

The DXA technique employs two distinct X-rays and is widely used to estimate fracture risk in the general population [24]. Although it remains inconclusive whether BMD measured by DXA effectively estimates fracture risk in patients undergoing hemodialysis [24], several observational studies have shown that BMD measured by the DXA method correlated with fracture risk in these patients [22, 25], suggesting that the DXA method, which can be easily performed in clinical settings, may help assess fracture risk in patients undergoing hemodialysis [26, 27]. Several clinical studies have reported that denosumab treatment is associated with maintaining or improving BMD at the femoral neck, lumbar spine, and distal radius [11]. Consistent with these findings, this study demonstrated long-term maintenance of BMD after denosumab administration, albeit specifically at the distal radius. However, measuring BMD at the distal radius is reportedly unsuitable for assessing the effects of denosumab [17], which is one of the limitations of this study. Although this study assessed BMD at the distal radius due to the facility and instrumentation, measuring BMD at the lumbar spine and femoral neck may have been ideal in terms of evaluating the effect of denosumab on BMD.

Hypocalcemia is a serious adverse effect denosumab and also occurs in patients with CKD, with an incidence rate of 15% [28]. The postulated mechanism parallels the dynamics of hungry bone syndrome observed after parathyroidectomy wherein the suppression of bone resorption induced by denosumab leads to an augmented Ca flux from the blood into the bone, culminating in acute hypocalcemia [29]. Prior studies have indicated that high baseline BAP and TRACP-5b levels are risk factors for hypocalcemia in patients undergoing hemodialysis based on the hypothesis that high bone turnover is associated with enhanced denosumab efficacy [16–18].

In this study, no significant associations were observed between hypocalcemia and BAP or TRACP-5b levels. Within the healthcare institution that facilitated this retrospective study, patients with a high risk of hypocalcemia attributable to denosumab were administered with prophylactic oral calcium supplementation. Such treatment might have confused the relationship between Ca and BTMs in this investigation. Hence, the findings of this study cannot conclusively rule out the potential link between baseline BTMs and hypocalcemia.

Although phosphate levels were the lowest on the seventh day after administration, they returned to baseline levels after 60 days and thereafter remained within the 3.5–6 mg/dL range, which is the recommended range by the Japanese CKD-MBD guidelines. [30] As in a previous report, [4] denosumab administration did not significantly change PTH levels, and there were no signs of changes in parathyroid function in the present study.

This study had several limitations. First, this study only included patients treated with denosumab, and a comparative reference group was lacking. Therefore, this study could not evaluate whether denosumab has therapeutic effects. Second, the difficulty in collecting longitudinal data on Ca supplementation and drugs that affect calcium, phosphate, and PTH levels, such as vitamin D analogs, prevented us from examining the effects of these changes on cCa levels after denosumab administration. Third, we could not analyze the occurrence of fractures due to the small number of patients and the inability to include a comparison group that did not receive denosumab. Fourth, only two points were used to assess BMD, and the possibility of measurement errors cannot be ruled out. Finally, the limited number of cases hindered the use of multivariate linear regression models to examine the association between BTMs and hypocalcemia, which led to analysis using scatter plots and regression lines. Future prospective and large-scale studies with whole-body BMD assessments are essential to elucidate the long-term effects of denosumab on the bone of patients undergoing hemodialysis.

In conclusion, the study showed long-term suppression of TRACP-5b and BAP levels and sustaining BMD after denosumab administration over an extended period in patients undergoing hemodialysis.

### Supplementary Information

Below is the link to the electronic supplementary material.

Supplementary file1 (PPTX 117 KB)

Supplementary file2 (PPTX 65 KB)

Supplementary file3 (PPTX 65 KB)
