# Supplementary material for: Long-term effects of denosumab on bone mineral density and turnover markers in patients undergoing hemodialysis
Source: J Bone Miner Metab. 2024 Mar 21;42(2):264–70. doi: 10.1007/s00774-024-01505-7 (PMC10982096; doi:10.1007/s00774-024-01505-7)
Supplement: Supplementary file 1 — Supplementary file1 (PPTX 117 KB) [file 774_2024_1505_MOESM1_ESM.pptx]

## Slide 1
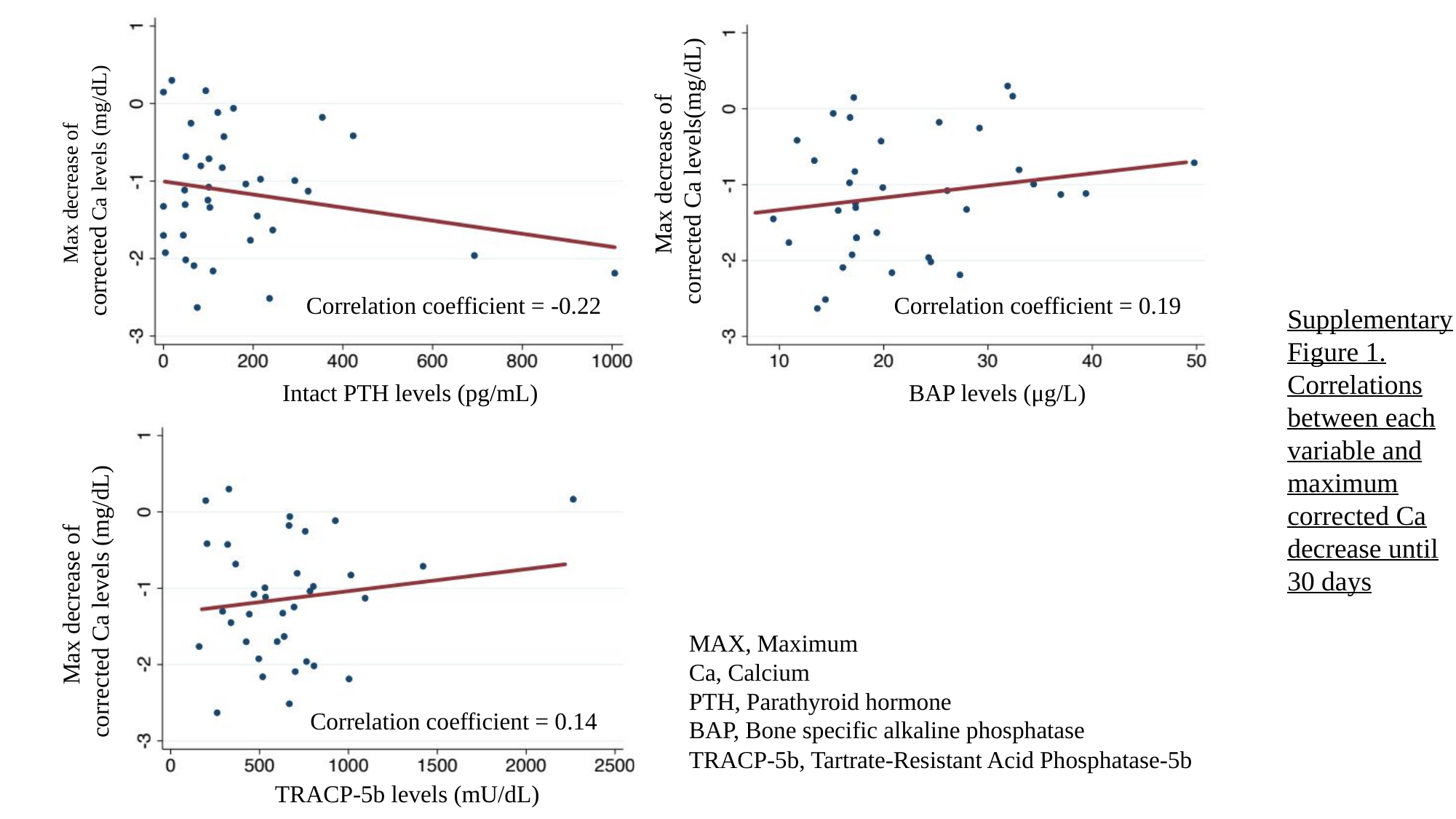

Max decrease of
corrected Ca levels(mg/dL)
Max decrease of
corrected Ca levels (mg/dL)
Correlation coefficient = -0.22
Correlation coefficient = 0.19
Supplementary Figure 1. Correlations between each variable and maximum corrected Ca decrease until 30 days
 Intact PTH levels (pg/mL)
BAP levels (μg/L)
Max decrease of
corrected Ca levels (mg/dL)
MAX, Maximum
Ca, Calcium
PTH, Parathyroid hormone
BAP, Bone specific alkaline phosphatase
TRACP-5b, Tartrate-Resistant Acid Phosphatase-5b
Correlation coefficient = 0.14
TRACP-5b levels (mU/dL)
