# Supplementary material for: Long-term effects of denosumab on bone mineral density and turnover markers in patients undergoing hemodialysis
Source: J Bone Miner Metab. 2024 Mar 21;42(2):264–70. doi: 10.1007/s00774-024-01505-7 (PMC10982096; doi:10.1007/s00774-024-01505-7)
Supplement: Supplementary file 2 — Supplementary file2 (PPTX 65 KB) [file 774_2024_1505_MOESM2_ESM.pptx]

## Slide 1
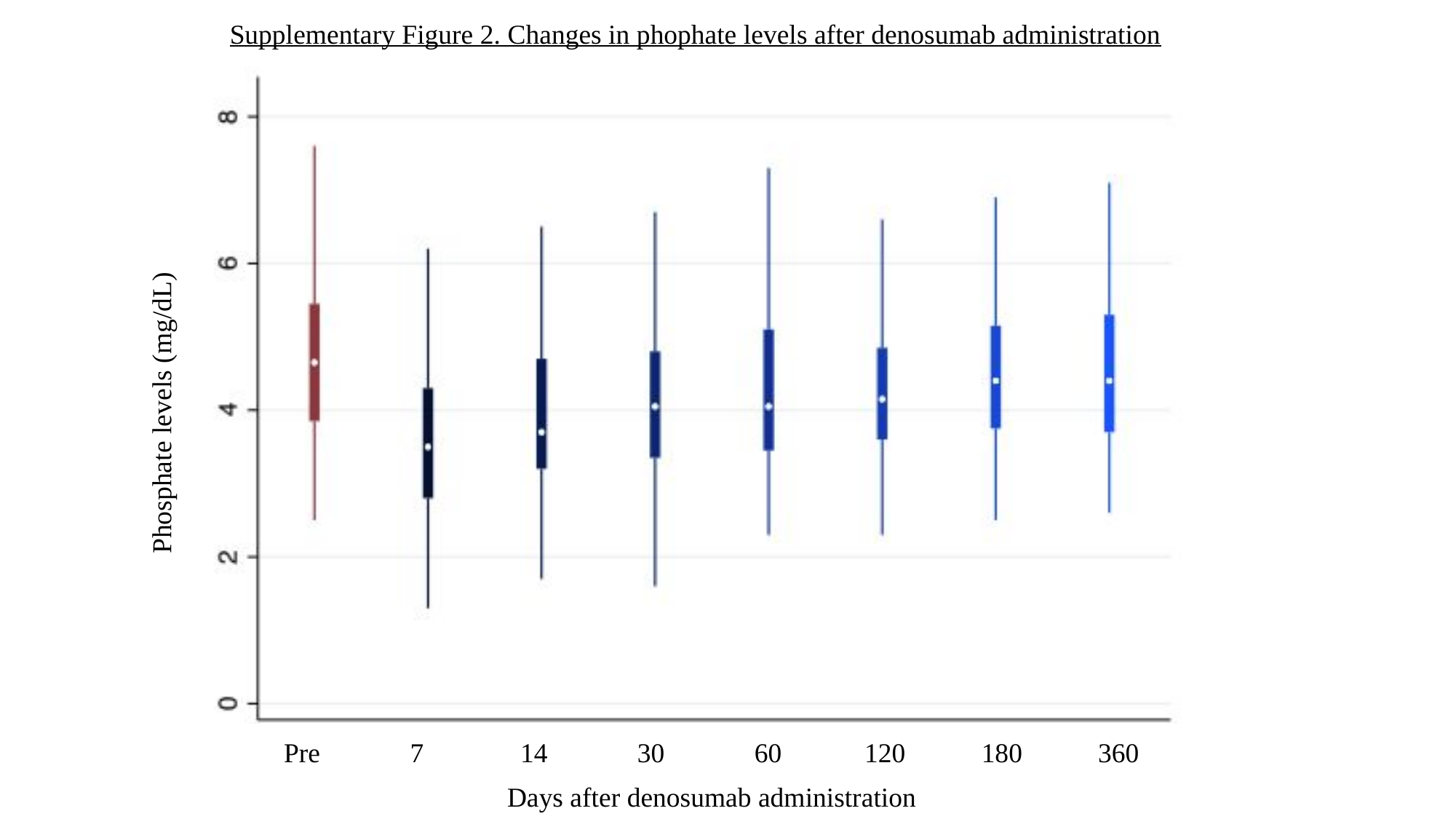

Supplementary Figure 2. Changes in phophate levels after denosumab administration
Phosphate levels (mg/dL)
Pre 7 14 30 60 120 180 360
Days after denosumab administration
