# Supplementary material for: Long-term effects of denosumab on bone mineral density and turnover markers in patients undergoing hemodialysis
Source: J Bone Miner Metab. 2024 Mar 21;42(2):264–70. doi: 10.1007/s00774-024-01505-7 (PMC10982096; doi:10.1007/s00774-024-01505-7)
Supplement: Supplementary file 3 — Supplementary file3 (PPTX 65 KB) [file 774_2024_1505_MOESM3_ESM.pptx]

## Slide 1
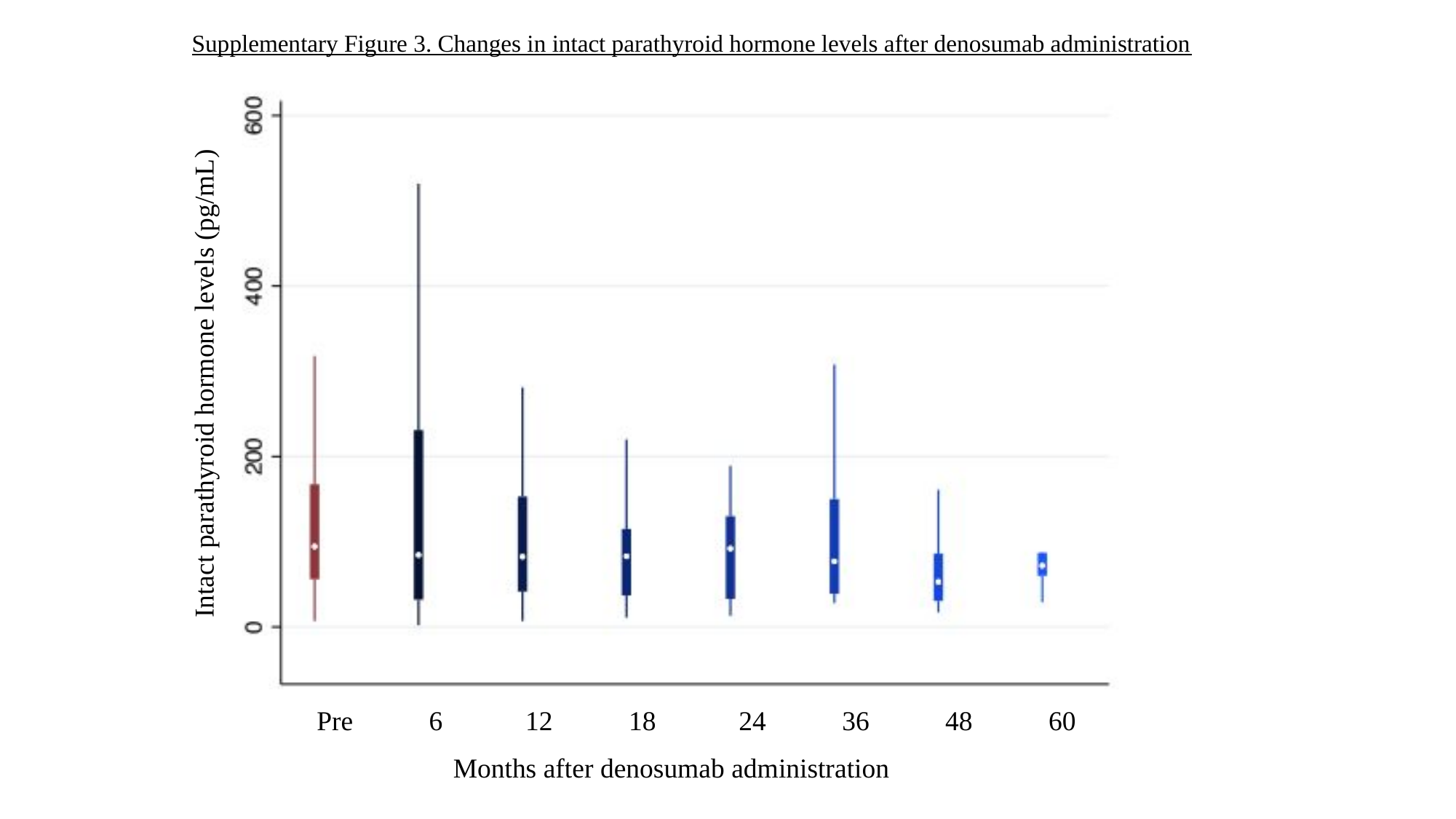

Supplementary Figure 3. Changes in intact parathyroid hormone levels after denosumab administration
Intact parathyroid hormone levels (pg/mL)
Pre 6 12 18 24 36 48 60
Months after denosumab administration
